# [^18^F]FDG, [^11^C]PiB, and [^18^F]AV-1451 PET Imaging of Neurodegeneration in Two Subjects With a History of Repetitive Trauma and Cognitive Decline

**DOI:** 10.3389/fneur.2019.00831

**Published:** 2019-08-02

**Authors:** David O. Okonkwo, Ross C. Puffer, Davneet S. Minhas, Sue R. Beers, Kathryn L. Edelman, Jane Sharpless, Charles M. Laymon, Brian J. Lopresti, Steven Benso, Ava M. Puccio, Sudhir Pathak, Milos D. Ikonomovic, Joseph M. Mettenburg, Walter Schneider, Chester A. Mathis, James M. Mountz

**Affiliations:** ^1^Department of Neurosurgery, University of Pittsburgh Medical Center, Pittsburgh, PA, United States; ^2^Department of Neurosurgery, Mayo Clinic, Rochester, MN, United States; ^3^Department of Radiology, University of Pittsburgh, Pittsburgh, PA, United States; ^4^Department of Psychiatry, University of Pittsburgh Medical Center, Pittsburgh, PA, United States; ^5^Learning Research and Development Center, University of Pittsburgh, Pittsburgh, PA, United States; ^6^Department of Neurology, University of Pittsburgh, Pittsburgh, PA, United States

**Keywords:** TBI, CTE, neurodegeneration, PET, PiB, amyloid, AV-1451, tau

## Abstract

**Background:** Trauma-related neurodegeneration can be difficult to differentiate from multifactorial neurodegenerative syndromes, both clinically and radiographically. We have initiated a protocol for *in vivo* imaging of patients with suspected TBI-related neurodegeneration utilizing volumetric MRI and PET studies, including [^18^F]FDG indexing cerebral glucose metabolism, [^11^C]PiB for Aβ deposition, and [^18^F]AV-1451 for tau deposition.

**Objective:** To present results from a neuroimaging protocol for *in vivo* evaluation of TBI-related neurodegeneration in patients with early-onset cognitive decline and a history of TBI.

**Methods:** Patients were enrolled in parallel TBI studies and underwent a comprehensive neuropsychological test battery as well as an imaging protocol of volumetric MRI and PET studies. Findings from two patients were compared with two age-matched control subjects without a history of TBI.

**Results:** Both chronic TBI patients demonstrated cognitive deficits consistent with early-onset dementia on neuropsychological testing, and one patient self-reported a diagnosis of probable early-onset AD. Imaging studies demonstrated significant [^18^F]AV-1451 uptake in the bilateral occipital lobes, substantial [^11^C]PiB uptake throughout the cortex in both TBI patients, and abnormally decreased [^18^F]FDG uptake in the posterior temporoparietal areas of the brain. One TBI patient also had subcortical volume loss. Control subjects demonstrated no appreciable [^18^F]AV-1451 or [^11^C]PiB uptake, had normal cortical volumes, and had normal cognition profiles on neuropsychological testing.

**Conclusions:** In the two patients presented, the [^11^C]PiB and [^18^F]FDG PET scans demonstrate uptake patterns characteristic of AD. [^11^C]PiB PET scans showed widespread neocortical uptake with less abnormal uptake in the occipital lobes, whereas there was significant [^18^F]AV-1451 uptake in both occipital lobes.

## Introduction

*In vivo* imaging characteristics of TBI-related neurodegeneration are currently being studied. Tau-specific positron emission tomography (PET) radiotracers being developed for Alzheimer's disease (AD) have shown promise in detecting tau pathology in patients who exhibit clinical signs of early cognitive impairment, including those with a history of TBI (1–3). [^18^F]FDG is a well-established PET radiotracer that measures neuronal cell metabolism and has a long history of use in the evaluation of brain metabolic reduction after TBI (4). In a recent study of a group of patients with a history of TBI, [^18^F]FDG PET demonstrated decreased uptake in clusters of brain voxels compared to normal controls (5). In addition, Pittsburgh compound B ([^11^C]PiB), an established amyloid-beta (Aβ) PET radioligand, was reported to have increased retention on PET imaging after TBI, the finding supported by postmortem [H-3]PiB autoradiography and Aβ immunohistochemistry in a separate cohort of TBI autopsy cases (6).

In pathology studies, the presence of diffuse Aβ plaques has been observed in resected brain tissue specimens from some severe TBI patients in acute injury phases, including patients of a relatively young age (7). Likewise, in autopsies of fatal-TBI victims, Chen et al. found amyloid plaques in subjects who died within hours of their injuries (8). However, they also found a complete absence of plaques in longer term survivors (27 days−3 years) despite evidence of neuronal Aβ production. Thus, the effect of TBI on AD risk is not clear.

Overlapping clinical and neuroimaging features of TBI- and AD- related cognitive deficits have been suggested to be attributable to synergy between the progression of chronic traumatic encephalopathy (CTE) and the development of early AD (9). Neuropathologic findings from two patients who developed early-onset dementia after moderate-severe Traumatic Brain Injury (TBI) support the hypothesis of post-TBI dementia as a polypathology with features that overlap with several dementia subtypes (10).

MRI evidence of gray matter volume loss is a structural feature common to many forms of dementia. Marketed software for the reproducible detection and analysis of regional brain volumes has shown utility in AD, TBI, and other neurodegenerative disorders (11–14).

We initiated a protocol for *in vivo* imaging of patients with TBI-related neurodegeneration utilizing volumetric MRI and PET imaging indices of cerebral glucose metabolism ([^18^F]FDG), Aβ burden ([^11^C]PiB), and tau burden ([^18^F]AV-1451). The objective of this case report is to present differences in these MRI and PET biomarkers in two patients with a history of chronic, repetitive TBI, one with predominantly blunt-force head trauma, and the other with a history of predominantly repetitive blast trauma.

## Methods

### Patient Selection

Both patients were identified after enrollment in research cohorts for studies approved by the Institutional Review Board at the University of Pittsburgh. Patients provided written informed consent for research participation and publication of this case report. Patient #1 was identified as a part of a cohort of patients being evaluated with advanced 3T MRI modalities for chronic TBI. Patient #2 was enrolled in the Targeted Evaluation Action and Monitoring of Traumatic Brain Injury (TEAM-TBI) trial, wherein patients undergo comprehensive clinical evaluations to adjudicate specific post-TBI sequelae. Two age- and gender-matched healthy controls were recruited for comparison.

Patient histories were obtained by trained interviewers and testing was completed by technicians supervised by the senior neuropsychologist (SRB). In both cases, chronic and acute symptoms and diagnoses following TBI were derived from patient-reported outcome measures (PROs) and a neuropsychological test battery. No formal medical record review was performed. Neuropsychological testing included measures of memory (California Verbal Learning Test [CVLT] Short and Long Delay Free Recall), executive function (Halstead Reitan Trail Making Test B [HRTMT-B), Wechsler Adult Intelligence Scale-IV [WAIS-IV] Working Memory Index [WMI], Controlled Oral Word Association Test [COWAT]), and information processing speed (WAIS-IV Processing Speed Index [PSI], HRTMT-A). Patient demographics and Neuropsychological test scores are shown in Table 1.

**Table 1:** Demographic comparisons.

	**Age**	**Gender**	**Ethnicity**	**Years education**	IQ estimate^*^	**Test validity**
Patient #1	60	M	Caucasian	17	101	Yes
Patient #2	54	M	Caucasian	12	092	Yes

* *IQ estimated with the North American Adult Reading Test (15)*.

All subjects underwent neuropsychological testing or an evaluation with the Mini-Mental Status Examination upon study entry (16). All neuroimaging studies were conducted over a 2-year period subsequent to neuropsychological testing.

### Imaging Protocol

Both patients and one control received structural T1-weighted MRI scans including a Magnetization Prepared Rapid Gradient Echo (MPRAGE) sequence on a 3T Siemens TIM Trio (Siemens Healthcare GmbH, Erlangen, Germany) scanner with a voxel size of 1 × 1 × 1 mm^3^. The second control received a structural T1-weighted MPRAGE sequence scan on a 3T Siemens TIM Trio with a modified voxel size of 1 × 1 × 1.2 mm^3^, both within recommended parameters for the volumetric software used.

[^11^C]PiB PET and [^18^F]FDG scans for each patient were performed on the same day using a Siemens ECAT Exact HR+ PET scanner (Siemens Healthcare GmbH, Erlangen, Germany). [^11^C]PiB scanning commenced 40 min after an average injection of 14.8 mCi. Data were acquired in 3D mode over 40–70 min post injection and binned into six 5-min frames. PET data were corrected for attenuation, scatter, and radioactive decay, converted to a 2D data set using Fourier rebinning, and reconstructed using the Direct Fourier (DIFT) method, similar to filtered back projection (FBP), into a 128 × 128 × 63 matrix with voxel sizes of 2.06 × 2.06 × 2.43 mm^3^. Images were filtered with a 3 mm Hann window.

[^18^F]FDG scanning commenced 35 min after an average injection of 7.6 mCi and were acquired over the interval 35–60 min post injection into five 5-min time frames. To minimize contamination of [^18^F]FDG data by residual ^11^C radioactivity, procedures were timed such that a minimum of 100 min (five ^11^C halflives) elapsed between the initial [^11^C]PiB injection and the start of the [^18^F]FDG PET scan. Reconstruction and data correction methods were similar to those used for [^11^C]PiB image processing.

On a different day, [^18^F]AV-1451 PET imaging was performed on a Siemens mCT-Flow Biograph PET/CT. Scanning commenced 75 min after an average injection of 9.1 mCi [^18^F]AV-1451. Data were binned into six 5-min frames over a 30 min scan. A low-dose, non-diagnostic CT without contrast was performed immediately before PET acquisition for use in attenuation and scatter correction. PET data were reconstructed via Fourier rebinning (FORE), followed by FBP using manufacturer's software into a 256 × 256 × 109 matrix with voxel sizes of 1.03 × 1.03 × 2.03 mm^3^. Reconstruction included corrections for scatter, attenuation, decay, random coincidences, and scanner deadtime. Images were filtered using a 3D 2 mm Hann window.

One control received only a [^18^F]AV-1451 PET scan, and the second control received only a [^11^C]PiB PET scan. All control and patient PET scans were conducted with identical protocols.

### Image Analysis

PET images were inspected and, if necessary, corrected for interframe motion using the interframe registration tool in the PFUS module of PMOD software (PMOD Technologies LLC, 2015, Zurich, Switzerland). Dynamic PET data were averaged over 40–60 min for [^18^F]FDG, 50–70 min for [^11^C]PiB, and 80–100 min for [^18^F]AV-1451, and each single-frame average PET image was independently registered to the patient's or control's corresponding structural MRI.

For qualitative visual reads of PET, MR images were normalized to Montreal Neurological Institute (MNI) template space using the Unified method within Statistical Parametric Mapping, version 12 (SPM12) software (17). The resulting transformation was applied to corresponding registered and averaged PET images. Cerebellar gray matter (GM) activity was sampled from each normalized PET using the Centiloid reference region (18), and standardized uptake value ratio (SUVR) images were generated by dividing each image by its cerebellar GM activity.

For regional quantification of PET, native-space MR images were processed through FreeSurfer v5.3 software (19). Standard FreeSurfer regions of interest were applied to corresponding PETs to sample radioactivity concentrations, and regional SUVR values were calculated using FreeSurfer's cerebellar cortex as reference.

Volumetric analysis was then performed on the MPRAGE images using NeuroReader (Brainreader, Horsens, Denmark) software (20). For each subject the analysis consisted of registering, segmenting, and measuring regional brain volumes. The regional volumes were corrected for age, gender, and total intracranial volume and compared to a normative control population, resulting in a percentile score.

## Results

### Patient #1

This 60-year-old Caucasian male (see Table 1) had a history of 45-year involvement in scholastic and collegiate athletics, including 30 years active participation, followed by 15 years of coaching. During his career, and starting in high school, he self-reported multiple head injuries, both sub-concussive and concussive. He specifically recalls an injury at 21 years of age where he sustained a significant blow to the head, with a post-concussive syndrome that caused him to be removed from practicing for a short period of time. He was never hospitalized or evaluated acutely after any of these injuries.

Shortly before he retired from coaching at age 54, he began to experience a precipitous decline in memory, mood swings, and cognitive difficulties. These symptoms were initially noted by his wife, and they included hypersomnia, irritability, difficulty concentrating, sensitivity to light and memory problems. He was evaluated by a neurologist and diagnosed with early-onset AD. There was no reported family history of early-onset AD. His neuropsychological testing (Table 2) demonstrated significant cognitive impairment across multiple test domains, including WAIS-IV Processing Speed Index (<1st percentile), WAIS-IV Working Memory Index (<1st percentile), Trail Making Test A (<1st percentile), and Trail Making Test B (unable to complete) (21).

**Table 2:** Neuropsychological test scores^*^.

	**Memory**		**Executive function**			**Information processing speed**	
	**CVLT SDFR**	**CVLT LDFR**	**WAIS-IV WMI**	**COWAT**	**Trails B**	**Trails A**	**WAIS-IV PSI**
Patient #1	<0.05	<0.05	0.04	UTC	UTC	0.01	<1
Patient #2	7	<1	18	<1	0.01	0.01	1

* *Scores represented as age corrected percentiles. CVLT, California Verbal Learning Test; SDFR, Short Delay Free Recall; LDFR, Long Delay Free Recall; WAIS-IV, Wechsler Adult Intelligence Scale-IV; WMI, Working Memory Index; COWA, Controlled Oral Word Association Test; PSI, Processing Speed Index (21); UTC, Unable to complete*.

**Figure 1:**
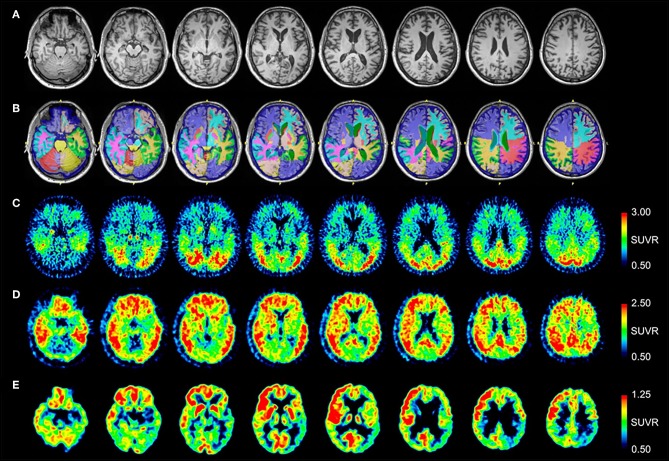
Serial transverse brain sections from **(A)** structural MRI, **(B)** NeuroReader segmentation, **(C)** [^18^F]AV-1451 PET, **(D)** [^11^C]PiB PET, and **(E)** [^18^F]FDG PET for Patient #1. The color bar shows uptake in standardized uptake value ratios (SUVR) with the cerebellar cortex as reference. **(A,B)** The MR image demonstrates evidence of decreased hippocampal volume. Occipital lobe volumes were normal. **(C)** The [^18^F]AV-1451 PET scan shows broad areas of abnormal intensely increased uptake in the cuneus (FreeSurfer mean [FS] SUVR: 2.39) and lateral occipital regions (FS SUVR: 2.28). There is also abnormally increased uptake in the superior parietal (FS SUVR: 2.19), inferior lateral temporal (FS SUVR: 1.79), and entorhinal brain regions (FS SUVR: 1.72). **(D)** The [^11^C]PiB PET scan demonstrates abnormally increased uptake in the temporal, parietal, and frontal lobes, as well as the precuneus, in a pattern characteristic of AD. Note that, by comparison, the occipital lobes have relatively less abnormal uptake. **(E)** The [^18^F]FDG scan shows abnormally decreased uptake in the posterior brain regions. The degree of asymmetry is much lower in the left posterior temporoparietal brain region, also a pattern characteristic of AD.

MRI and PET imaging results are shown in Figure 1. Quantitative analysis of the MRI scan demonstrated hippocampal and amygdala volumes to be low (left hippocampus−2.74 ml, 11th percentile; right hippocampus−2.86 ml, 12th percentile; left amygdala−0.78 ml, 14th percentile; right amygdala−0.83 ml, 12th percentile). Left temporal lobe volume was also found to be low (left temporal lobe−99.10, 19th percentile; right temporal lobe−114.26 ml, 43rd percentile). All other cerebral cortex volumes, including the occipital lobe, were normal (left occipital 59.17 ml, 69th percentile; right occipital 50.35 ml, 50th percentile). Selected NeuroReader-derived regional volumes and percentiles are presented in Table 3. The [^18^F]AV-1451 PET scan showed broad areas of abnormal intensely increased [^18^F]AV-1451 uptake, primarily in the medial and lateral occipital regions, but also in the parietal regions. There was also abnormally increased uptake in the mesial temporal structures. The [^11^C]PiB PET scan demonstrated abnormally increased uptake in the temporal, parietal, and frontal lobes, as well as the precuneus, in a pattern characteristic of AD, while the occipital lobes had relatively less abnormal uptake. The [^18^F]FDG scan showed abnormally decreased uptake in the posterior brain regions. In summary, while the [^11^C]PiB and [^18^F]FDG PET scans had typical AD patterns, the [^18^F]AV-1451 appeared more typical for posterior cortical atrophy (PCA) syndrome, a form of dementia that is usually considered an atypical variant of AD (22–24).

**Table 3:** NeuroReader regional brain MRI volumetric results.

	**Patient #1 ml (percentile)**	**Patient #2 ml (percentile)**	**Control #1 ml (percentile)**	**Control #2 ml (percentile)**
Left hippocampus	2.74 (11.07)^*^	4.12 (50.87)	3.59 (35.45)	4.13 (52.69)
Right hippocampus	2.86 (12.77)^*^	4.25 (51.33)	3.49 (30.75)	4.41 (60.51)
Left amygdala	0.78 (14.72)^*^	1.39 (46.46)	1.63 (58.65)	1.98 (84.06)
Right amygdala	0.83 (12.04)^*^	1.37 (42.34)	1.78 (63.84)	2.13 (90.56)
Left frontal lobe	209.93 (47.48)	221.24 (59.77)	218.99 (59.38)	230.36 (78.97)
Right frontal lobe	217.64 (56.30)	211.33 (53.37)	217.87 (59.68)	224.87 (73.51)
Left parietal lobe	96.39 (32.18)	106.63 (51.07)	110.73 (57.25)	99.61 (37.82)
Right parietal lobe	101.09 (40.03)	94.42 (36.45)	109.44 (57.27)	102.17 (44.41)
Left occipital lobe	59.17 (69.47)	52.43 (54.49)	60.65 (73.27)	50.83 (48.82)
Right occipital lobe	50.35 (50.83)	50.31 (54.47)	54.21 (63.77)	55.38 (71.36)
Left temporal lobe	99.10 (19.37)^*^	123.60 (59.33)	115.36 (48.60)	116.94 (53.59)
Right temporal lobe	114.26 (43.30)	116.56 (50.42)	117.89 (52.11)	112.87 (43.64)

* *Regional volume with a percentile lower than 25% relative to a normative population after correcting for age, gender, and total intracranial volume*.

### Patient #2

This 54-year-old Caucasian male trained in explosive ordinance disposal and served for 26 years on active military duty, including multiple deployments to Iraq and Afghanistan. He was employed a further 7 years as a contractor to a private defense company that focused on training bomb defusing teams. He reported at least 5 episodes of concussive injury to the head over his career, a TBI episode resulting in loss of consciousness in a parachuting accident in 2000, as well as exposure to hundreds of subconcussive blasts while acting as a team leader in Afghanistan defusing improvised explosive devices (IED). In 2007, he was involved in an IED attack where he was exposed to the blast while riding in a vehicle. He self-reported a slow deterioration in his cognition and memory beginning the year after the IED attack in 2007. Specifically, he reported significant memory difficulties and manifested a tremor in his hands. Self-reported symptoms were confirmed with his spouse due to his difficulty with memory.

As a part of his enrollment in a research trial, he underwent a neuropsychological test battery (Table 2) demonstrating significant cognitive impairment, including WAIS-IV Processing Speed Index (1st percentile), Trail Making Test A (<1st percentile), and Trail Making Test B (<1st Percentile).

**Figure 2:**
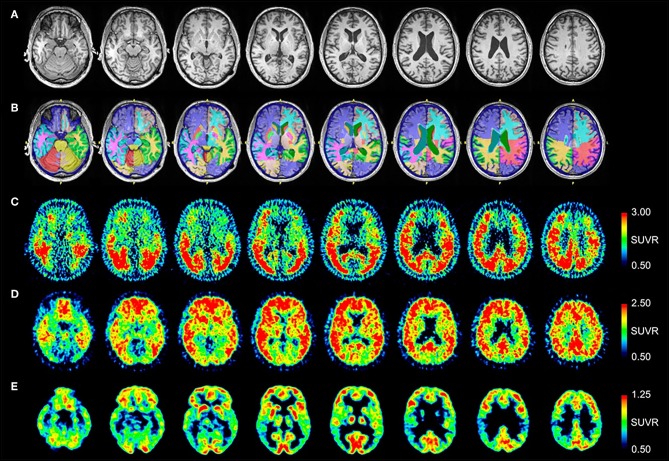
Serial transverse brain sections from **(A)** structural MRI, **(B)** NeuroReader segmentation, **(C)** [^18^F]AV-1451 PET, **(D)** [^11^C]PiB PET, and **(E)** [^18^F]FDG PET for Patient #2. **(A,B)** Quantitative analysis of the MRI scan demonstrated normal volumes. **(C)** The [^18^F]AV-1451 PET scan is most significant for demonstrating abnormal intensely increased symmetric uptake in the precuneus (FreeSurfer mean [FS] SUVR: 2.97), superior parietal (FS SUVR: 2.86), and lateral occipital regions (FS SUVR: 2.76). There is also abnormally increased uptake in the lateral inferior temporal (FS SUVR: 2.47), entorhinal (FS SUVR: 2.13), lateral orbitofrontal (FS SUVR: 1.78), and superior frontal (FS SUVR: 1.67) regions. **(D)** The [^11^C]PiB PET scan demonstrates significant abnormally increased uptake in parietal, temporal and frontal lobes, as in Patient #1, demonstrating a pattern characteristic of AD. Note there is negligible accumulation in the occipital lobes (as compared to the [^18^F]AV-1451 scan). **(E)** The [^18^F]FDG PET scan shows abnormally and relatively symmetric decreased uptake in the posterior temporoparietal regions of the brain, with sparing of the frontal lobes.

MRI and PET results are shown in Figure 2. Quantitative analysis of the MRI scan demonstrates normal hippocampal, amygdala, and cerebral cortex volumes (Table 3). The [^18^F]AV-1451 PET scan demonstrates abnormal, intensely increased symmetric uptake primarily in the parietal and lateral occipital regions. There is also abnormally increased uptake in the orbitofrontal, posterior frontal, lateral temporal, and mesial temporal brain regions. The [^11^C]PiB PET scan demonstrates abnormally increased uptake in parietal, temporal and frontal lobes, as in Patient #1, a pattern characteristic of AD. Note there is negligible accumulation in the occipital lobes (as compared to the [^18^F]AV-1451 scan). The [^18^F]FDG PET scan shows abnormally and relatively symmetric decreased uptake in the posterior temporoparietal regions of the brain, with sparing of the frontal lobes. In summary, the [^18^F]AV-1451, [^11^C]PiB, and [^18^F]FDG PET scans demonstrate typical AD patterns of uptake.

### Controls

**Figure 3:**
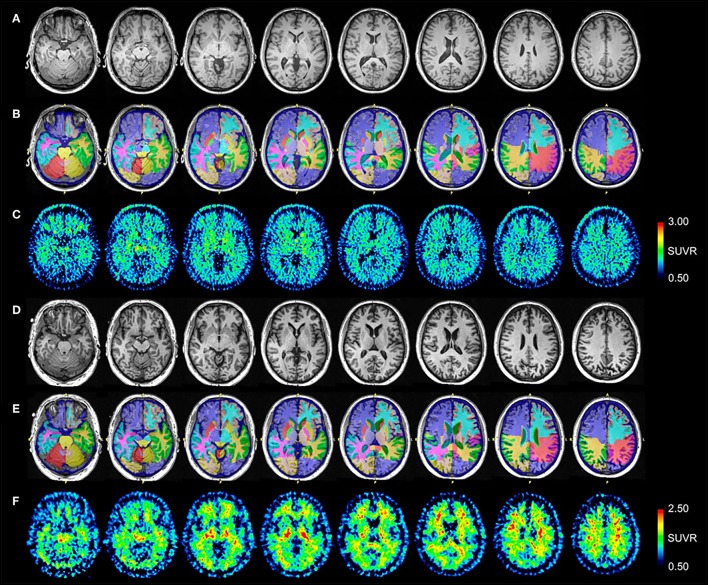
Serial transverse brain sections from **(A)** structural MRI, **(B)** NeuroReader segmentation, and **(C)** [^18^F]AV-1451 PET scans for Control #1; and **(D)** structural MRI, **(E)** NeuroReader segmentation, and **(F)** [^11^C]PiB PET scans for Control #2. SUVR color bar scales for each PET radiotracer are identical across all patients and controls. The [^18^F]AV-1451 image for Control #1 shows mild basal ganglia, substantia nigra, and choroid plexus uptake that reflects a typical pattern of off-target binding. By comparison with both patients, there is negligible occipital, frontal, temporal, and parietal lobe uptake. The [^11^C]PiB image for Control #2 shows non-specific uptake in white matter regions, but negligible uptake throughout neocortical and subcortical gray matter.

Control #1 is a 58-year-old male and Control #2 is a 64-year-old male. Neither control had a significant history of TBI or neurodegenerative symptoms. Control #1 scored within normal limits on all testing for the neuropsychological battery administered to patients. Control #2 scored 30/30 on the MMSE. Volumetric analysis demonstrated no abnormalities in any brain region. Normalized MRI, NeuroReader segmentation, and [^18^F]AV-1451 SUVR images for Control #1 are presented in Figures 3A–C, respectively. Normalized MRI, NeuroReader segmentation, and [^11^C]PiB SUVR images for Control #2 are presented in Figures 3D–F, respectively.

## Discussion

In this case series, we report neuroimaging and clinical findings in two patients with neurodegenerative disease and a history of multiple concussive and subconcussive brain injuries. Neither case reported a family history of early-onset dementia. Both patients had severe neuropsychological impairment as demonstrated on dedicated testing. We utilized an imaging protocol that included volumetric MRI and PET studies including: [^18^F]FDG metabolism; [^11^C]PiB, an Aβ specific PET tracer; and [^18^F]AV-1451, a tau PET tracer. Both patients demonstrated decreased [^18^F]FDG metabolism in posterior brain regions and global increased uptake of [^11^C]PiB similar to that demonstrated in AD (25). The pattern of [^18^F] AV-1451 uptake is most significant in occipital, parietal, and temporal lobes in both patients. This pattern is similar to the uptake pattern of other tau-specific radiotracers that have been studied in patients with suspected CTE (1, 2). While earlier CTE neuropathology stages spare the occipital lobes, global, non-selective tau deposition can be seen in stage IV disease (26). It has previously been reported that predominant PCA with tau deposition can be seen in individuals thought to have a specific phenotype of AD selectively involving the posterior cortical regions, highlighting the potential difficulties of diagnosing trauma-related neurodegeneration from variants of AD using PET radiotracers specific for amyloid and tau (22–24). Neither of our patients, however, demonstrated appreciable occipital lobe atrophy on volumetric analysis, despite the substantial tauopathy and FDG hypometabolism on PET imaging.

It is currently unknown if *in vivo* PET and structural MR imaging demonstrating significant PCA with tau deposition is found commonly in patients with AD and a co-existent history of TBI. However, it has been observed that onset of PCA generally occurs earlier than in typical AD, with most patients experiencing first symptoms in their 50s or early 60s (27). It should be noted that [^18^F]AV-1451 and other putative tau ligands still require postmortem validation to determine specific pathological substrates for their binding *in vivo*, due to the complexity of tau isoforms, post-translational modifications, and neuropathological aggregates in AD and non-AD tauopathies including CTE (28).

The binding of various tau tracers to different tau isoforms could explain observations of tracer-dependent cortical distributions (29–31). It is possible the posterior cerebral degeneration pattern seen here may reflect the binding of [^18^F]AV-1451 to one or several tau isoforms or conformations arising from the combined effects of trauma and more intrinsic predilection for development of AD. Nevertheless, the pattern of uptake demonstrated in the patients presented here is not unique to our experience, and similar patterns have previously been reported in suspected CTE and typical and atypical variants of AD (1, 2, 22–24).

This study is limited by its small subject sample and retrospective nature, making it difficult to report significant associations or the predictive ability of these imaging techniques when it comes to *in-vivo* diagnosis of either trauma-related neurodegeneration or multifactorial neurodegenerative syndromes. In addition, there are no diagnostic standards associated with [^18^F]AV-1451. Thus, the imaging studies presented here should not be viewed as a clinical evaluation and were not performed with diagnostic intent. Instead, our purpose is to use these data to guide evaluation of a larger cohort of patients with suspected traumatic encephalopathy with the goal of validating an *in-vivo* diagnostic panel for trauma-related neurodegeneration.

## Conclusions

Structural T1-weighted MRI and [^18^F]FDG, [^11^C]PiB, and [^18^F]AV-1451 PET hold promise as neuroimaging biomarkers for detecting trauma-related neurodegeneration in living patients with a history of traumatic brain injuries; however, there may be overlap with other neurodegenerative syndromes potentially unrelated to trauma, and distinction between these conditions will require further study.

## Data Availability

The datasets generated for this study are available on request to the corresponding author.

## Ethics Statement

This study was carried out in accordance with the recommendations of the Institutional Review Board of the University of Pittsburgh. The protocol was approved by the Institutional Review Board.

## Informed Consent

All subjects gave written informed consent for research participation and publication of this case report in accordance with the Declaration of Helsinki.

## Author Contributions

DO, DM, SRB, KE, JS, CL, SB, AP, SP, MI, JMe, WS, CM, and JMo contributed to the conception and design of the study. DM, CL, SP, JMe, and JMo performed image and statistical analyses. RP wrote the first draft of the manuscript. DO, DM, SRB, CL, BL, MI, JMe, and JMo wrote sections of the manuscript. All authors contributed to manuscript revision, read and approved the submitted version.

### Conflict of Interest Statement

CM has licensed intellectual property with GE Healthcare. GE Healthcare provided no grant support for this study and had no role in the design or interpretation of results or preparation of this manuscript. The remaining authors declare that the research was conducted in the absence of any commercial or financial relationships that could be construed as a potential conflict of interest.
